# Horns on the Human Subject

**Published:** 1830-10-01

**Authors:** 


### Horns on the Human Subject.

A commission of the Royal Academy of Medicine of Paris has been considering the subject of human horns—we do not mean those with which satirists deck us, but *bonâ* fide genuine horns. The commission have collected from various authors so many as 71 recorded cases; 31 in men, 37 in women, and 3 in young children. In 9 cases they were on the head, in 3 on the forehead, in 12 on the thigh, in 3 on the temple, in 5 on the nose, in 2 on the cheek, in 1 on the jaw, in 4 on the chest, in 4 on the back, in 3 on the penis and glans, in 1 on the ischium, in 2 on the knee and calf, in 1 on the leg, and in 2 on the feet. In a young woman whose case is related by Bonnet, horns grew so fast from every part of the body, more particularly the joints, that at thirteen years of age she was covered with them. Some of these productions were twisted like ram's horns, and when any fell off, others grew in their place. A horn two or three inches in length grew from the end of each finger—a formidable appendage, we imagine, to a lady. The commission think, with M. Breschet, that the skin and the mucous membrane are the only textures in the human body that form horns. M. Virey considers them as warts exceedingly enlarged from their being situated so as to receive large vessels, but this is manifestly absurd. We believe that these horny projections very frequently form from the sebaceous glands. These become enlarged, their secretion is locked up within them and becomes indurated, ulceration of the surface of the cyst then takes place, and the contained hard, horny secretion is thrust out through the opening. This at least is not unfrequently the case in the head, and we believe that Sir Everard Home removed such an ornamental projection from the head of an old woman in St. George's Hospital.

